# Mechanical Properties of Cement-Based Gel Composites Reinforced by Plant Fiber: A Review

**DOI:** 10.3390/gels11050362

**Published:** 2025-05-14

**Authors:** Peng Zhang, Xiao Zhang, Jinjun Guo, Yuanxun Zheng, Zhen Gao

**Affiliations:** 1 School of Water Conservancy and Transportation, Zhengzhou University, Zhengzhou 450001, China; zhangpeng@zzu.edu.cn (P.Z.);; 2 State Key Laboratory of Tunnel Boring Machine and Intelligent Operation, Zhengzhou 450001, China

**Keywords:** plant fiber, cement-based gel composites, modification methods, mechanical properties, interfacial bonding properties

## Abstract

Plant fibers (PFs) have been increasingly employed in cement-based gel composites (CCs) on account of their excellent mechanical properties, toughness and sustainability. Researchers have engaged in a lot of studies on plant fiber-reinforced cement-based gel composites (PFRCCs). Based on these studies, the chemical components and mechanical characteristics of PFs are summed up in this review. In addition, the modification methods for matrices and PFs are also discussed. The mechanical properties of PFRCCs, including static and dynamic properties, are reviewed. Predictive equations for the mechanical properties of PFRCCs are summarized in this paper. In the end, the characteristics of the interface transition zones between PFs and CCs are analyzed. According to the results of previous studies, the addition of PFs can enhance the flexural strength and tensile strength of CCs, but it can have an uncertain effect on compressive strength. The elastic modulus and fracture behavior of PFRCCs increases with the addition of PFs. At the same time, modification methods have been proved to be useful in reducing the degradation of PFs in CCs. Generally speaking, PFRCCs are new building materials which have extensive application prospects. The aim of this review is to help researchers understand the mechanical properties of PFRCCs and to promote the application of PFRCCs in future projects.

## 1. Introduction

Cement-based gel composites (CCs), such as concrete, mortar and paste, are widely used in construction industry nowadays. However, the fabrication of cement is complex and environmentally harmful. There are three stages of cement production, including raw material preparation, heat treatment and fabrication of cement [1]. The primary raw material of cement is limestone, which has an extensive source. The pyro-processing stage, encompassing preheating (800–900 °C), pre-calcination (~30% decarbonation efficiency), clinker sintering in rotary kilns (1450 °C peak temperature) and rapid quenching via grate coolers, accounts for 85–90% of the total thermal energy consumption in conventional Portland cement manufacturing [2]. The environmental pollution caused by cement production is extremely high and is very significant compared with other industries. According to statistics, in China, the cement industry accounts for 14.8%, 12.3%, 26.2% and 2.4% of the country’s total emissions of carbon dioxide, nitrogen oxides, particulate matter and sulfur dioxide, respectively [3]. Although CCs have been widely employed in building engineering, there are still some defects of CCs that need to be noted. The first type of defect of CCs is the existence of pores in the matrices, which harm the mechanical properties and durability of cement. Recent research has revealed that porosity is highly correlated with mechanical properties [4,5]. The correlation coefficients between porosity and compressive strength and tensile strength are −0.9428 and −0.9146, respectively [6]. The other defect is that CCs exhibit poor strain capacity [7]. It is necessary to improve the toughness of CCs. A widely adopted strategy to enhance the mechanical performance of CCs involves the incorporation of supplementary materials, including nanomaterials, discontinuous fibers and polymeric binders [8,9,10]. Fiber-reinforced cement-based gel composites (FRCCs) have advantages in terms of mechanical properties, shrinkage and expansion resistance [11]. It should be noted that the content and type of fibers within CCs are key parameters with respect to their rheological properties, while the rheological properties of FRCCs are responsible for the orientation and dispersion of fibers [12]. Extensive incorporation of fibers often leads to agglomeration, which makes casting and mixing of FRCCs increasingly difficult [13]. For this reason, the appropriate fiber type and content need to be controlled during the fabrication process of FRCCs.

**Figure 1:**
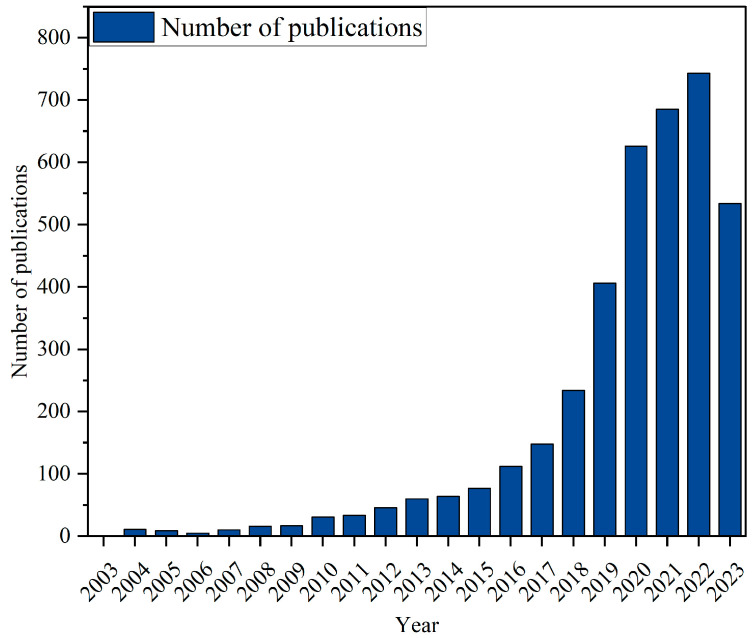
Numbers of publications about PFRCCs.

Steel, glass, basalt, polypropylene and other fibers are frequently utilized to reinforce CCs [14,15,16,17]. However, these synthetic fibers have their own limitations. Recent research indicated that the diffusion coefficient of chloride ions in mortars with steel fibers was two to three times higher than without steel fibers [18], which suggests that the addition of steel fiber in CCs will increase the corrosion risk. According to the research of Silva et al. [19], glass fiber has little effect on ductility. Meanwhile, the thickening of polypropylene fiber in CCs affects their compaction properties [20]. Even though basalt fibers exhibit outstanding durability and mechanical qualities, their uneven dispersion restricts their widespread application in CCs [21]. In addition, all the aforementioned fibers rely on raw material resources, which contribute to increased production costs [22]. One primary problem of adding traditional fibers in CCs is that it causes pollution and waste reuse issues [23]. One solution to this problem is to use plant fiber (PF) instead of traditional fibers, such as steel fibers and synthetic fibers [24]. Commonly used PFs, such as sisal fibers, jute fibers, hemp fibers, flax fibers and coconut fibers, exhibit good water absorption, degradability and stable mechanical properties [25,26,27,28,29,30,31,32,33,34,35]. As shown in Figure 1, the interest in utilizing PF in CCs is steadily increasing. This phenomenon is mainly attributed to the demand for environmental protection. PFRCCs can significantly reduce the utilization of natural resources and have a relatively long life cycle [36]. Due to these advantages, research on the application of PF in CCs has garnered growing interest. The increasing use of PF is due to PF exhibiting the ability to enhance the flexural and tensile properties [37,38], elastic moduli and dynamic mechanical properties [39] of CCs. On the one hand, the use of PF instead of traditional fiber materials can reduce carbon emissions and production costs [40]. On the other hand, PF can enhance the rheology behavior, mechanical properties (including static and dynamic mechanical properties), ductility and toughness of CCs [41,42]. For example, the incorporation of coconut fiber in CCs has been shown to significantly decrease yield stress, which demonstrates improvement in workability [43]. Recent studies have demonstrated that incorporating jute fiber into CCs can significantly enhance the compressive strength of jute fiber-reinforced CCs [44], and the flexural strength of CCs can be promoted with the addition of hemp fiber [28]. Moreover, some studies have focused on PF’s ability to accelerate cement hydration at the early stage and reduce shrinkage [45]. However, PF has a low elastic modulus [46]. Consequently, the compressive strength of plant fiber-reinforced cement-based gel composites (PFRCCs) may not satisfy the requirements for engineering applications. A number of works have shown that this problem can be solved by using modification methods (e.g., alkali treatment, acetylation treatment, oxide treatment, thermal treatment, etc.). Fiber modification and matrix modification represent two distinct categories of modification techniques [47,48].

A comprehensive review of the existing literature will improve the comprehension of the mechanical characteristics of PFRCCs. Consequently, this review summarized the mechanical properties of PFRCCs, drawing upon findings from previous research. The chemical compositions and mechanical properties of PFs commonly used in CCs were reviewed. Then, the degradation mechanism of PFs in CCs as well as various types of modification measures were reviewed. After that, the mechanical properties of PFRCCs were reviewed, including compressive properties, flexural properties, tensile properties, moduli of elasticity, fracture performance and dynamic mechanical properties. In addition, the interfacial bonding properties of PFRCCs were also reviewed in this paper. The aim here is to summarize the mechanical properties of PFRCCs and provide a theory for the further application of PFs in CCs.

## 2. Characteristics of PF

### 2.1. Chemical Composition of PF

**Figure 2:**
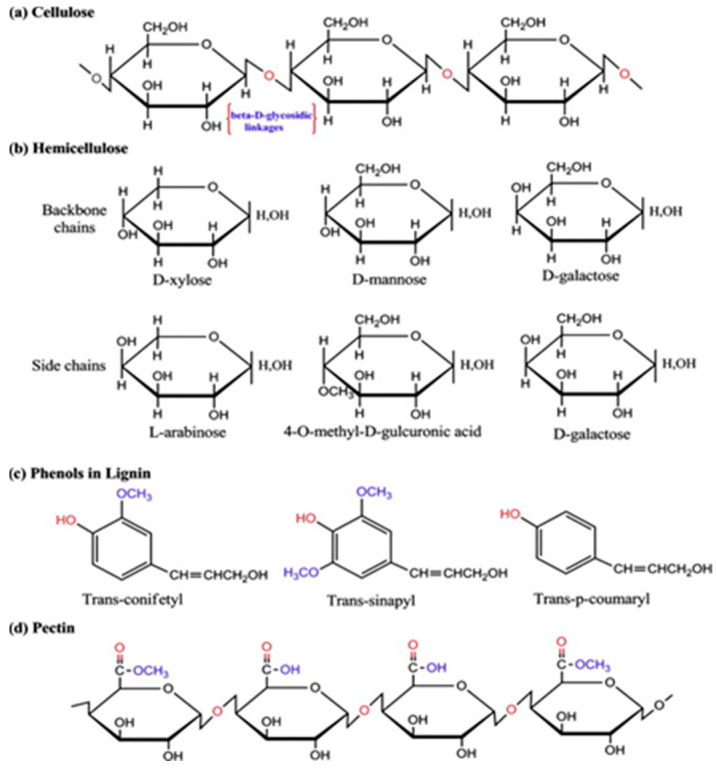
Chemical composition of plant fiber [51].

The four principal constituents of PF are generally recognized as cellulose, hemicellulose, lignin and pectin [49,50]. The chemical structures of cellulose, hemicellulose, pectin and lignin are shown in Figure 2 [51]. The physical and mechanical properties of PF are mainly determined by its chemical composition. In PF, cellulose is recognized as the most robust and resilient organic component, significantly contributing to the strength, rigidity and stability of the plant structure [52]. Cellulose molecules are glucose units arranged together by bundles of microfibers [52]. Cellulose is a semi-crystalline polysaccharide characterized by a substantial presence of hydroxyl groups, which confer hydrophilic properties to the molecule [53]. This hydrophilicity results in poor interfacial bonding when plant fiber is used as a reinforcement in CCs [54]. Hemicellulose is a polysaccharide linked together in relatively short branch chains, closely related to cellulose microfibers [52]. Hemicellulose is intricately associated with cellulose fibrils through the formation of hydrogen bonds [55]. Lignin is an amorphous three-dimensional polymer consisting of phenylpropane units which are crosslinked by C-C bonds and ether bonds [56]. Lignin gives plants rigidity; without lignin, plants cannot grow very tall. The shine of plants is mainly determined by pectin. Moreover, pectin can act as a bonding agent between microfibers. Table 1 lists the chemical components of some common PFs. Furthermore, the chemical composition of PFs is influenced by various factors, including species, geographical conditions and climatic factors, all of which subsequently affect their performance characteristics [57].

### 2.2. Common PFs in Construction Engineering

**Figure 3:**
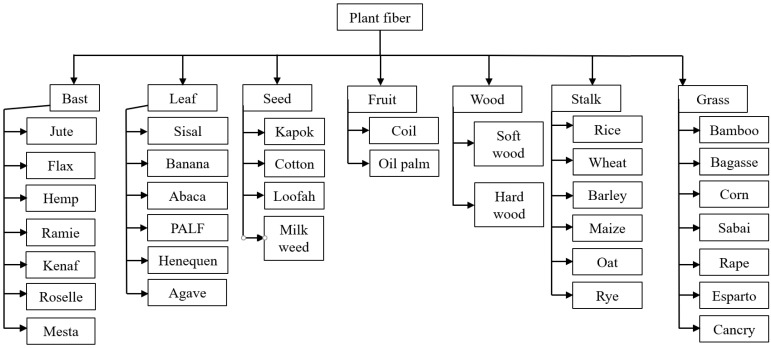
Classification of PFs [66].

**Figure 4:**
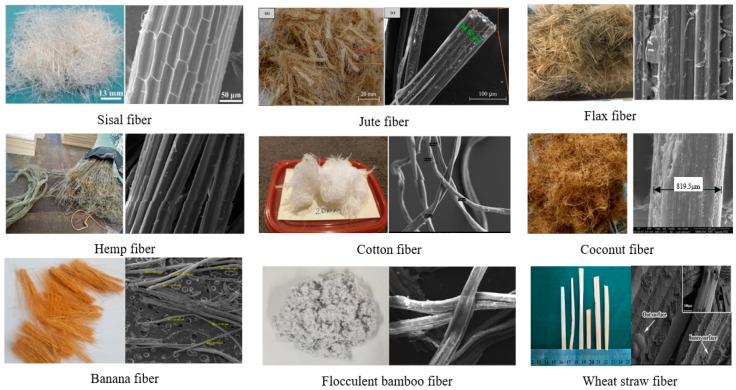
Macroscopic and microscopic photographs of PFs utilized in cement-based gel composites [31,67,68,69,70,71,72,73].

PFs are classified as natural fibers; a classification is shown in Figure 3. Figure 4 presents several macroscopic and scanning electron microscope (SEM) images of PFs utilized in construction applications. The microstructure of a PF is porous, and the surface is rough [51]. Jute fiber, flax fiber and hemp fiber have similar microstructures because they come from the bast of plants [66]. The microtopography of sisal fiber has obvious stripes, which help it to bond with a cement matrix [67]. Among a variety of PFs, sisal fiber, flax fiber, hemp fiber, jute fiber and cotton fiber are currently used in construction engineering.

**Figure 5:**
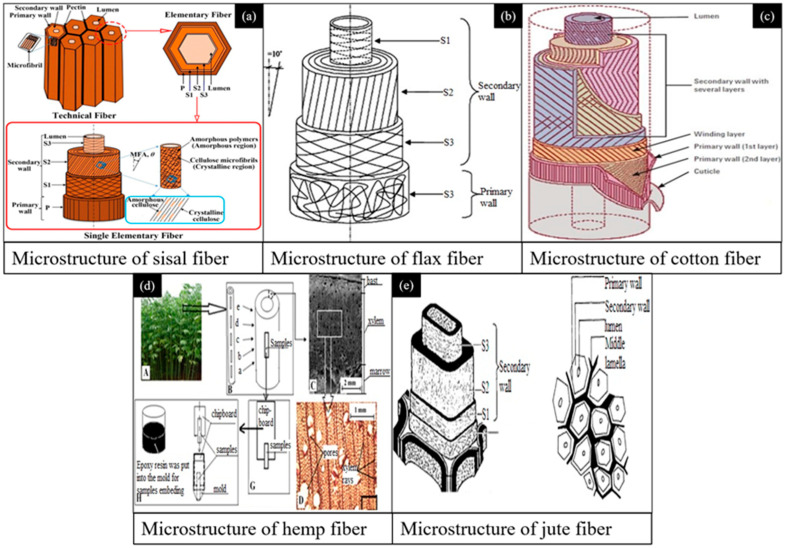
Microstructures of different plant fibers, including (**a**) sisal fiber, (**b**) flax fiber, (**c**) cotton fiber, (**d**) hemp fiber and (**e**) jute fiber [52,77,78,79,80,81].

Figure 5 presents microstructure diagrams of sisal fiber, flax fiber, cotton fiber, hemp fiber and jute fiber. It is obvious that they exhibit similar microstructures, which are made of two walls (a primary wall and a secondary wall) [60]. Their similar microstructures give PFs similar mechanical properties. In general, PFs exhibit a hierarchical architecture comprising helically oriented, concentric cell walls, where each layer demonstrates distinct compositional and microstructural configurations [74]. As illustrated in Figure 5, the constituent layers exhibit variable thicknesses and spatial organizations, with a central lumen surrounded by progressively developed cellular strata [75]. A predominant secondary cell wall forms the outermost structural component, encapsulating both the primary cell wall and the central lumen through its substantial thickness. The mechanical performance of a PF is principally governed by the cellulose concentration and the preferential orientation of microfibrils along the cellular longitudinal axis, which directly correlates with its tensile strength and structural rigidity [76].

Cellulose nanofibers, representing an advanced developmental stage of PF utilization, exhibit markedly enhanced interfacial adhesion with cement matrices due to their high specific surface areas, which promote matrix densification, thereby yielding superior mechanical homogeneity and performance metrics [82,83]. While nanocellulose fiber applications have predominantly been confined to polymer matrices in the past, their incipient integration into cementitious systems has been increased. The incorporation of cellulose nanofibers has two distinct morphological classifications: cellulose nanocrystals synthesized via acid hydrolysis and cellulose nanofibrils—entangled networks produced through high-shear mechanical fibrillation—each demonstrating unique reinforcement mechanisms in calcium silicate hydrate (C-S-H)-dominated matrices [84,85].

### 2.3. Physical and Mechanical Properties of PFs

The mechanical and physical properties of PFs significantly influence the performance of PFRCCs when they are employed as reinforcement materials in CCs. Table 2 lists the mechanical properties of plant fibers commonly used in the literature [52].

The primary factors influencing the strength of PFRCCs are the tensile strength and elongation properties of the plant fibers [44]. A PF with a high tensile strength serves to improve the bridging effect between the PF and the CC [44]. In addition, the different manufacturing processes used in various studies can also lead to differences in test results. For this reason, standards or specifications should be proposed according to the characteristics of the extraction processes and the test methods of PFs, their composition and the local climate.

## 3. Modification Methods

Reducing the degradation of PF in a cement matrix is a huge challenge in promoting the widespread application of PF. Consequently, it is essential to employ modification techniques to enhance the durability of PF and address the challenges associated with insufficient interfacial bonding, fiber degradation within the matrix and the high water-absorption characteristics of PFRCCs [86]. This section provides an overview of the degradation mechanism of PF in cement matrices and the modification methods.

### 3.1. Degradation Mechanisms of PF in Cement Matrices

Cementitious systems—encompassing paste, mortar and concrete—exhibit inherent alkalinity due to portlandite (Ca(OH)_2_) formation during hydration, with initial pore solution pH values typically ranging from 12.5 to 13.8 [87]. Notwithstanding the significant efficacy of PFs as reinforcing agents in improving the mechanical properties of CCs, their deterioration in the alkaline environment of cement substantially limits their wide application [88]. How to solve the degradation of PFs is a major challenge for the practical application of PFs. The primary factor contributing to the degradation of PFs is the alkaline environment created by Ca(OH)_2_, which is produced during the hydration process of cement [89]. When PFs are exposed to alkaline pore solutions in a cement-based gel matrix, degradation occurs due to alkaline hydrolysis [90].

**Figure 6:**
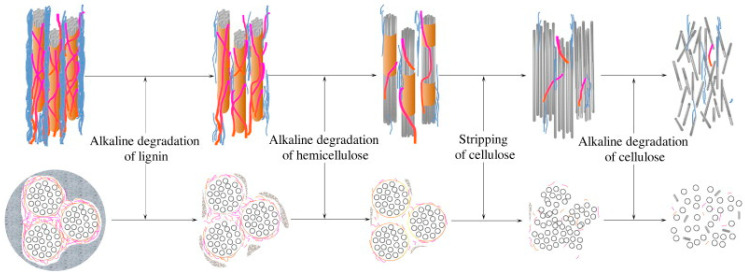
Degradation processes of PFs in cement matrices [93].

The degradation process begins with the swelling of PFs, which in turn leads to microcracks in the matrix surrounding the swollen fibers [91]. The main types of plant fiber degradation are mineralization, biodegradation and degradation due to alkali attack [92]. As illustrated in Figure 6, there are four phases in the degradation of PFs in cement matrices. The initial phase involves the breakdown of lignin and hemicellulose, which subsequently exposes cellulose in the matrix. The second stage is mainly the degradation of cellulose, which leads to a decrease in the stability of the plant cell wall. The third stage involves the distribution of microfibrils in the alkaline matrix, a process that subsequently enhances the degradation of cellulose. The final phase involves the disruption of cellulose fibril filaments, which occurs due to alkaline hydrolysis affecting the amorphous region surrounding the non-reducing end. This process ultimately leads to the complete degradation of plant fiber [93].

Alongside the alkaline hydrolysis of the three primary components, the mineralization of the cell wall serves as a significant mechanism contributing to the embrittlement of PF, as well as a decrease in both strength and ductility [94]. Research indicates that the mineralization of cell walls is influenced by the concentration of Ca(OH)_2_. Cement hydration—an exothermic reaction series initiated by the interaction of Portland cement (C_3_S, C_2_S, C_3_A and C_4_AF) with water—progressively transforms the fresh paste into a hardened matrix through the formation of calcium silicate hydrate (C-S-H) gel, portlandite (Ca(OH)_2_) and ettringite (AFt). This microstructural evolution governs the development of the pH and dimensional stability in cementitious systems [95]. Ca(OH)_2_ mineralization and self-mineralization are two types of mineralization mechanisms. The movement of Ca(OH)_2_ and Ca^2^⁺ into the lumen of a PF contributes to the degradation of the PF [95]. Self-mineralization serves as an indicator of the hydrolysis rate of amorphous components, suggesting that the alkalinity of the matrix may accelerate the degradation of PFs [93]. In research on the influence of alkaline environments on PFs, PFs were soaked in two alkaline solutions of sodium hydroxide with different pHs for one week [96]. This research was conducted to simulate the alkaline degradation of PFs within a cement matrix. The findings indicate that the degradation of PFs negatively impacts the mechanical properties of PFRCCs [96].

### 3.2. Cement-Based Gel Material Modification

Ordinary Portland cement (OPC) is widely used as a binder material in concrete production. It is important to recognize that the traditional cement manufacturing process significantly contributes to environmental pollution [97]. Therefore, other environmentally friendly materials should be sought to replace cement, such as fly ash (FA), silica fume (SF), biotite, etc. The simultaneous use of PF and other environmentally friendly materials is highly effective in promoting the recycling of resources. This section describes the properties of PFRCCs containing volcanic ash material. Numerous studies have shown that the addition of these materials can enhance the mechanical and durability properties of PFRCCs, as shown in Table 3.

**Figure 7:**
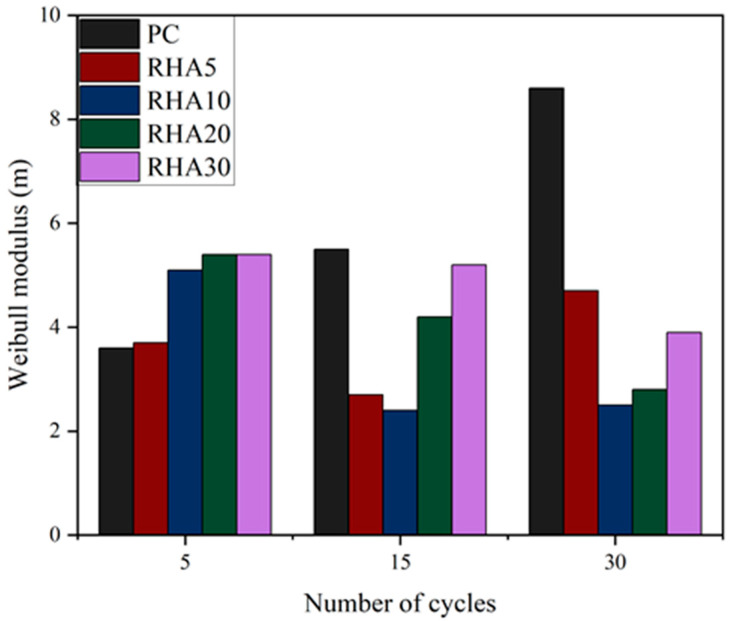
Effects of number of wet and dry cycles and rice husk ash dosage on Weibull moduli of PFRCCs [101].

Adding fly ash to CCs has various effects; for example, it can increase the durability of concrete. Meanwhile, the use of fly ash reduces the use of cement, which can reduce project costs and provide a suitable material for structural applications [98]. Compared with plain concrete, adding fly ash to concrete makes the matrix density higher and the porosity between the matrix and the fiber lower, which in turn results in more overall fiber-to-matrix contact points [99]. Calcium sulfa-aluminate cement maintains the long-term toughness of flax fiber concrete [100]. The degree of hydration, the concentration of calcium hydroxide and the alkalinity of the cement matrix are critical factors that influence the alkali-induced degradation of the cell walls of plant fibers. Incorporation of rice husk ash (RHA) in the substrate can effectively reduce the degradation of sisal fiber. After 30 wet and dry cycles, a 30% dosage of RHA showed a significant reduction in the Weibull modulus, which to some extent proves the beneficial role of rice husk ash in mitigating the degradation of sisal fibers, as shown in Figure 7 [101]. Partial replacement of silicate cement with different supplementary cement-based gel materials, such as biotite, fly ash and crumb, can also reduce the degradation of PF in cement-based gel composites [102].

**Table 3:** Impact of matrix modification on mechanical and durability characteristics of PFRCCs.

Modification Materials	Dosages	Fiber	Mechanical Characteristics	Durability	Ref.
FA and waste glass (WGs)	15% FA replacement of cement; 14%, 15%, 16%, 17%, 18%, 19% and 20% WG replacement of sand	Coconut	16% WG, compressive strength: 47.2 MPa, flexural strength: 6.2 MPa	Water absorption: 2.25, 14% WGs Penetration height: 58 mm, 20% WGs	[47]
Metakaolin	15% and 30% replacement of cement	Flax	15%, compressive strength: 35.4 MPa 15%, flexural strength: 6.5 MPa	15%, pH: 12.26; 30%, pH: 12.20	[100]
Ground-granulated blast furnace slag (GGBS)	30% and 60% replacement of cement	Flax	30%, compressive strength: 28.4 MPa; 30%, flexural strength: 5.6 MPa	30%, pH: 12.26; 60%, pH: 12.17	[100]
Calcium sulfa-aluminate cement (CŠA)	100% replacement of cement	Flax	Compressive strength: 25.8 MPa Flexural strength: 5.6 MPa	pH: 10.32	[100]
Rice husk ash	30% equal weight replacement cement	Sisal	-	Ultimate tensile strength: 384% ↑ (after 30 wet–dry cycles)	[101]
SF	5%, 10%, 15% and 20% replacement of cement	Coconut	Compressive strength: 25% ↑ Compressive pre-crack energy absorption: 71% ↑ Flexural strength: 34% ↑ Flexural post-crack energy absorption: 105% ↑	-	[103]
Alkali-activated material	100% replacement of cement	Wheat straw	Flexural strength: 18% ↑	Permeability: 12% ↓	[104]

### 3.3. Fiber Modification

#### 3.3.1. Chemical Modification of PF

Chemical modification of PF is used to enhance fiber-to-matrix bonding and reduce moisture transfer [91]. The objective of chemical treatment is to modify the chemical composition or surface characteristics of PF, thereby facilitating the attainment of a stable structure with improved bonding properties [105]. Common methods of chemical modification of PF are shown in Table 4, including alkali treatment, acetylation treatment, Na_2_CO_3_ treatment and oxide treatment [27,35,89,90,106,107,108,109,110,111,112]. Chemical pretreatment technology can be used to treat impurities (fats, waxes and minerals) on the surface of PF to enhance fiber properties.

##### Alkali Treatment

**Figure 8:**
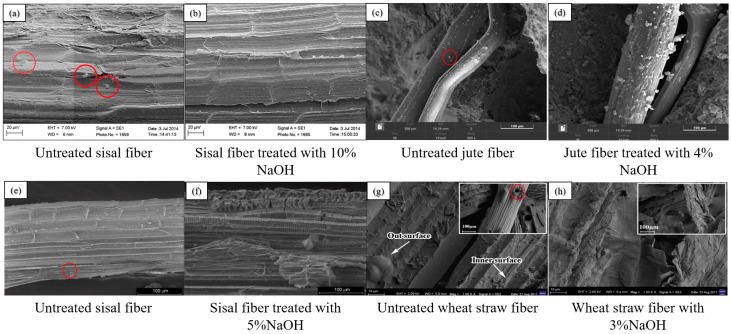
Comparison of SEM images of the surfaces of sisal, jute and straw fibers after alkali treatment: (**a**) Untreated sisal fiber; (**b**) Sisal fiber treated with 10% NaOH; (**c**) Untreated jute fiber; (**d**) Jute fiber treated with 4% NaOH; (**e**) Untreated sisal fiber; (**f**) Sisal fiber treated with 5% NaOH; (**g**) Untreated wheat straw fiber; (**h**) Wheat straw fiber with 3% NaOH [27,73,89,114].

Eliminating hemicellulose and lignin from PF cell walls while reducing the quantity of hydrophilic hydroxyl groups is the aim of alkali treatment [118]. In addition, the increase in surface roughness of PF is attributed to the deposition of calcium, which leads to the degradation of hydroxyl groups within the PF [114]. SEM images of alkali-treated PF indicate a reduction in surface defects, suggesting that this treatment enhances the potential for effective interfacial bonding with CCs, as shown in Figure 8 [27,73,89,114].

**Figure 9:**
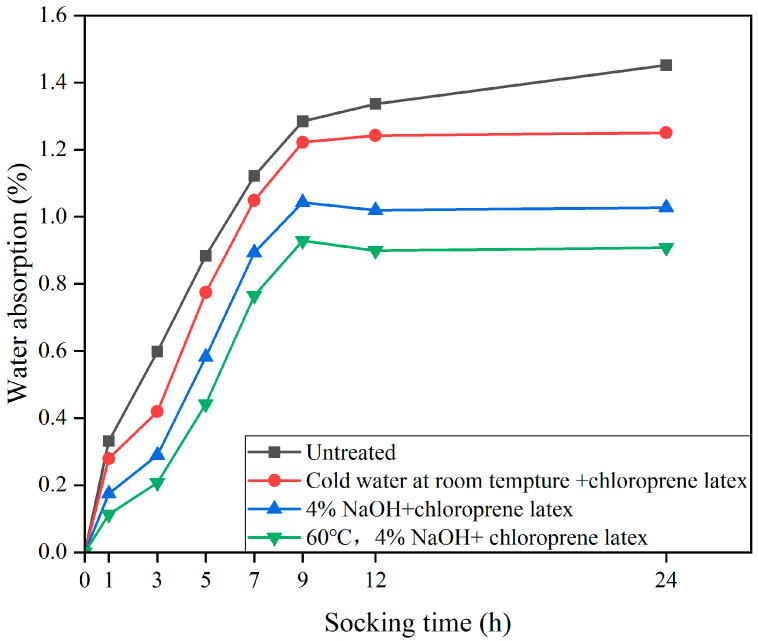
Impact on water absorption rates of different groups after soaking in chloroprene latex solution [27].

In fact, alkali treatment also affects the water absorbency of jute fibers, as shown in Figure 9 [27]. When these textured fibers were immersed in chloroprene rubber emulsion, the microscale surface roughness facilitated mechanical interlocking with the polymer matrix. This interaction promoted the formation of a continuous hydrophobic film on the fiber surface. This phenomenon indicates that alkali treatment can lead to the degradation of cellulose and hemicellulose on the surface of PF [27]. This is supported by Jiang et al. [73], who argue that under alkaline conditions, hemicellulose and lignin contents are hydrolyzed and dissolved into resins and glucuronic acids. Dissolution of hemicellulose and lignin results in a loose porous structure that increases the water absorption of PF.

##### Acetylation Treatment

Acetylation treatment can remove the impurities on the surface of PF, thereby decreasing the degradation rate of PF in a cement matrix [119]. In the research of Klerk et al. [114], 10% acetic anhydride and 5% acetic acid were used to acetylate sisal fibers. Acetic acid and acetic anhydride were adopted for sisal fiber immersion, both for one hour. Subsequently, the fibers were extracted from the solution and subjected to a washing process with water to eliminate any residual acid on their surfaces. Ultimately, the fibers were shaped in an unfolding condition and subsequently underwent 2–3 days of maintenance until their weight was stable [114]. The experimental findings indicated that the fibers subjected to acetic acid treatment were pulled out and broken when they were buried at 15 mm and 20 mm. In contrast, the pull-out of fibers with the acetic anhydride treatment was presented at a 10 mm embedding depth, whereas the fracture of fibers occurred at a 15 mm embedding depth [114]. This indicates that PF treated with acetic anhydride exhibits robust adhesion with the matrix.

##### Na_2_CO_3_ Treatment

Na_2_CO_3_ is also used for the modification of PF. Wei and Meyer modified sisal fibers using Na_2_CO_3_ solution at concentrations of 7% and 10% [90]. The experimental findings indicated a significant increase in the Young’s modulus of the sisal fibers, with a remarkable enhancement of 37% observed after a soaking period of 7 days, followed by an additional increase of 12% after 10 days of soaking. The surface microstructure of the sisal fibers immersed for seven days showed no significant changes. Following a 10-day immersion period, there was a significant increase in the surface roughness of the fibers. After Na_2_CO_3_ treatment, the crystallinity of the fibers increased from 20.27% to 22.69% [90]. Equations (1) and (2) show the chemical reactions that occurred when the sisal fibers were soaked in saturated Na_2_CO_3_ solution and then added to fresh concrete [90]. The protective layer formed by calcium carbonate protects the fibers from strong alkali solutions during cement hydration and fills in depressions in the sisal fiber surface [117].

(1) $$Ca{\left(OH\right)}_{2}\to Ca^{2+}+2OH^{-}$$

(2) $$Ca^{2+}+CO_{3}^{2-}\to CaCO_{3}↓$$

##### Oxide Treatment

**Figure 10:**
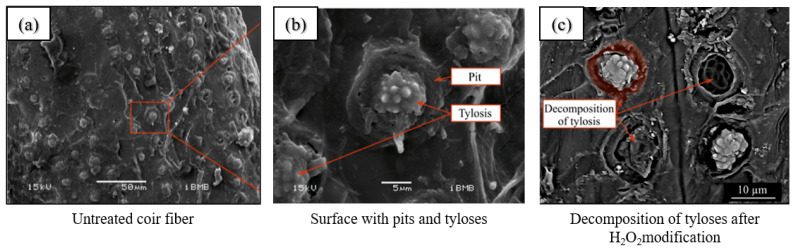
SEM images of the surface of coconut fiber after oxide treatment: (**a**) Untreated coir fiber; (**b**) Surface with pits and tyloses; (**c**) Decomposition of tyloses after H2O2 modification [118].

The application of oxide treatment to PF has been demonstrated to alter the surface characteristics of the fibers, thereby improving the adhesion between the fibers and the matrix. The researchers modified coir fibers using 5% H_2_O_2_ and then immersed the modified coir fibers in Ca(OH)_2_ for 12 weeks and performed tensile tests at intervals of 0, 4 and 12 weeks [118]. Thermogravimetric analysis and Fourier transform infrared spectroscopy (FT-IR) provided evidence of the decomposition of hemicellulose in the coir fibers, as well as the formation of CaCO_3_ on the surface of the fibers when exposed to Ca(OH)_2_ solution [118]. From the SEM images, it can be seen that among all the modification methods, only the modification with H_2_O_2_ solution revealed a unique change in the fiber surface. As shown in Figure 10, many fibers were disintegrated and gaps could be found between the caseous areas and the pits (red-marked portions), which was not the case for the unmodified fibers [118]. The findings subsequent to the encapsulation of ZrO_2_ and the impregnation of flax fibers utilizing the sol–gel dip-coating technique indicated an approximate 40% enhancement in the contact angle of the treated flax fibers compared to their untreated counterparts [120]. This phenomenon can be attributed to the dense ZrO_2_ coating, which facilitates the interaction between water molecules and the cellulose surface.

#### 3.3.2. Physical Modification of Plant Fiber

##### Thermal Treatment

The thermal treatment of PF can rearrange and reorient cellulose microfibers and cause changes in the crystallinity fraction of the PF, which will enhance the stiffness of the PF [121]. One heat treatment operation involved heating PF in boiling water at a temperature of about 70 °C for 1 h. The fibers were then thermally treated at 150 °C for 8 h. Subsequent to the thermal treatment, the fibers were subjected to a cooling process and subsequently stored in self-sealing plastic bags, which were sealed to inhibit moisture absorption [120]. The thermally treated fibers were mixed into concrete at concentrations of 0.5%, 1.0%, 1.5% and 2.0% and tested for their mechanical properties. In comparison to the control group, the incorporation of heat-treated sisal fiber at concentrations of 0.5%, 1.0% and 1.5% into the concrete mix enhanced both compressive and tensile strength. However, it was noted that the flexural strength was reduced [117]. The increase in compressive strength was simply due to the sisal fibers being less likely to expand the microcracks formed due to loading, making the specimens more ductile compared to the control group [112]. The improvement in tensile strength can be attributed to the fibers’ ability to bridge across cracks [122]. The reduction in flexural strength can be attributed to the increase in porosity resulting from the incorporation of fibers [123].

#### 3.3.3. Hybridization Methods for PF

Fiber hybridization has been proved to be an effective method of combining the positive qualities of various fibers [124]. Carbon fibers and steel fibers are common employed in fiber hybridization with PF [125,126]. Carbon fibers have an excellent mixing effect with PF because of their hydrophobicity, which improves the workability of mixtures [125]. The hybridization of steel fibers and PF exhibits a huge increase in the mechanical properties of FRCCs, which is attributed to the high elastic modulus of steel fibers [126].

## 4. Mechanical Properties of PFRCCs

### 4.1. Compressive Behavior of PFRCCs

**Figure 11:**
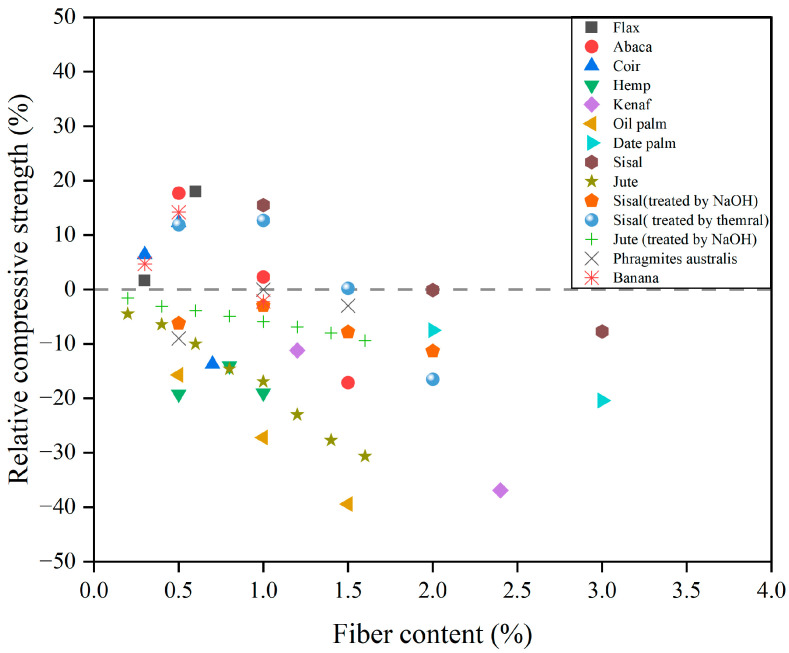
Impact of plant fiber on relative compressive strength of PFRCCs.

Compressive strength is an essential parameter used to determine the load capacity of a CC. CCs exhibit high compressive strength and can withstand great pressure [125]. The compressive load capacity of FRCCs is influenced by several factors, including fiber content, the ratio of fiber length to diameter and the geometric configuration of the fibers [103]. As shown in Figure 11, many studies have shown that the addition of PF can negatively affect the compressive strength of PFRCCs [27,33,64,65,127,128,129,130,131].

**Figure 12:**
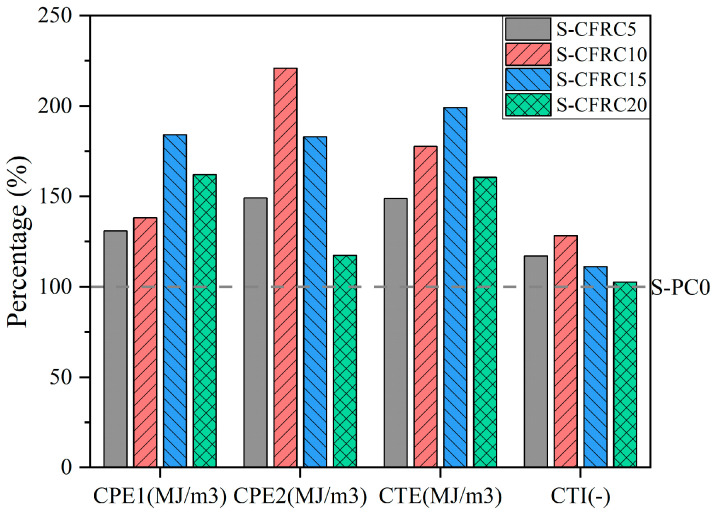
Effect of dosage of SF on CPE1, CPE2, CTE and CTI of CFRC.

The primary explanation of this phenomenon is the low strength of PF. At the same time, due to the moisture absorption rate and non-standard size, the adaptability between PF and cement matrices is relatively low, resulting in the formation and propagation of cracks in cement matrices [88]. Modification of a cement matrix or PF enhances the bonding properties of a PFRCC; this in turn enhances the compressive strength of the PFRCC [27,103]. As shown in Figure 12, with the addition of silica fume to a matrix, the pre-compression crack energy uptake (CPE1), post-compression crack energy uptake (CPE2), total compression energy uptake (CTE) and compression toughness indices (CTI) of coconut fiber-reinforced concrete (CFRC) were all improved across the board [103]. The indexes mentioned (CPE1, CPE2, CTE, CTI and CFRC) were based on the work of Khan et al. [132]. S-CFPC5, S-CFRC10, S-CFRC15 and S-CFRC20 represent the use of SF to replace cement at 5%, 10%, 15% and 20% substitution rates, respectively [103]. In addition, the research about the durability of the PFRCC revealed that the loss rate of compressive strength with age presented the durability of the PFRCC. According to the research of Raut and Gomez [33], compared to the compressive strength of palm oil fiber-reinforced CCs at 28 days, the compressive strength decreased by 37%. This is supported by Wang et al. As shown in Equation (6), the uniaxial compressive strength (UCS) exhibited a negative correlation with curing age [34].

Sultana et al. formulated a mathematical model to examine the relationship between fiber length (L), fiber volume (V), the water–cement ratio (W/C) and the compressive strength of jute fiber-reinforced concrete (JFRC). Using response surface analysis and crow search algorithms, the researchers accurately predicted the effect of jute fiber incorporation on the tensile properties of the JFRC, as shown in Equation (3) [133]. Another mathematical model based on an artificial neural network was proposed to predict the compressive strength of jute and coconut fiber-reinforced composites, as shown in Equations (4) and (5) [134]. Wang et al. obtained the relationship between UCS, age of maintenance and corn stover fiber content using Design-Expert software, as shown in Equation (6) [34].

(3) $$\begin{array}{l} f_{c}^{\prime }=28.05+0.732L-3.464V-0.055W/C-0.523L^{2}+\\ 0.758V^{2}+0.604W/C^{2}+1.697LV-2.654LW/C+0.709VW/C \end{array}$$

where *f_c_*′ is the compressive strength of the JFRC.

(4) $$\begin{array}{l} f_{cs14}=26.90+0.31X_{1}+0.11X_{3}-2.83X_{2}-2.56X_{3}^{2}-1.39X_{2}^{2}\\ +0.24X_{2}X_{3} \end{array}$$

(5) $$\begin{array}{l} f_{cs28}=31.64+0.268X_{1}-0.6X_{3}-3.02X_{2}-2.61X_{3}^{2}-1.51X_{2}^{2}\\ +0.41X_{2}X_{3} \end{array}$$

where *f_cs_*_14_ and *f_cs_*_28_ are compressive strengths at 14 days and 28 days and *X*_1_, *X*_2_ and *X*_3_ are jute, coconut fiber content and quarry dust, respectively.

(6) $$\begin{array}{l} UCS=7.96+1.4t-2.72\omega -1.77\omega t-2.4t^{2}+0.54\omega^{2}\\ +1.26t^{2}\omega +0.94\omega^{2}t+1.54t^{3}-0.16\omega^{3}(R^{2}=0.9795) \end{array}$$

where *t* is the curing age and *ω* is the fiber content.

From the above studies, it is evident that there exists an optimum amount of plant fiber incorporation in cement-based gel composites. The recommended dosages of different kinds of PFs are shown in Table 5. Excessive fiber incorporation often leads to agglomeration of fibers, which increases porosity and decreases the compressive strength of the PFRCC.

### 4.2. Flexural Behavior of PFRCCs

**Figure 13:**
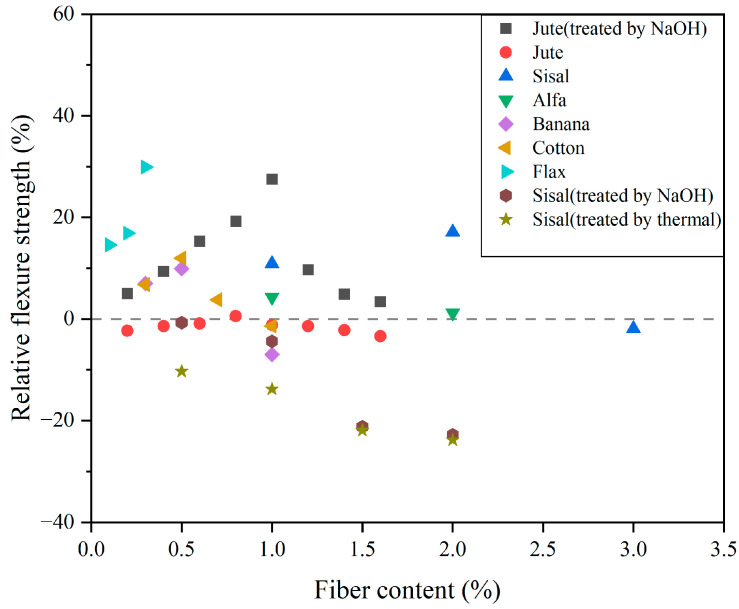
Effect of PF on the relative flexural strength of PFRCCs.

Flexural strength is typically characterized as the capacity of a CC to withstand bending loads. Crack-tip deformation can be categorized into three fundamental modes, the opening mode, the in-plane shear mode and the out-of-plane mode [135]. Due to its limited tensile strength and fracture toughness, a CC is prone to brittle failure. Incorporating PF into the matrix is a typical method to improve the flexural properties of PFRCCs [136]. This method improves bending behavior during strain hardening and multiple cracking. The incorporation of an appropriate dosage of PF into a CC demonstrates favorable post-peak flexural strength characteristics [137]. Due to the incorporation of PF, a specimen can maintain good shape integrity under large deformation. As shown in Figure 13, there exists an optimal dosage of PF [27,29,65,106,138,139,140,141]. Flexural strength tends to be negatively impacted when extensive fibers are mixed in.

**Figure 14:**
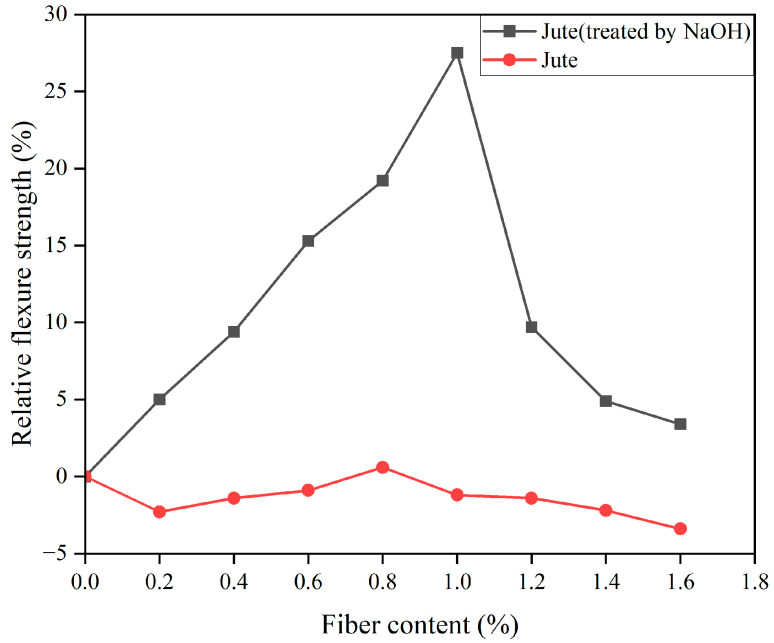
Impact of jute fiber treated with alkali and untreated jute fiber on the relative flexural strength of JFRC.

**Figure 15:**
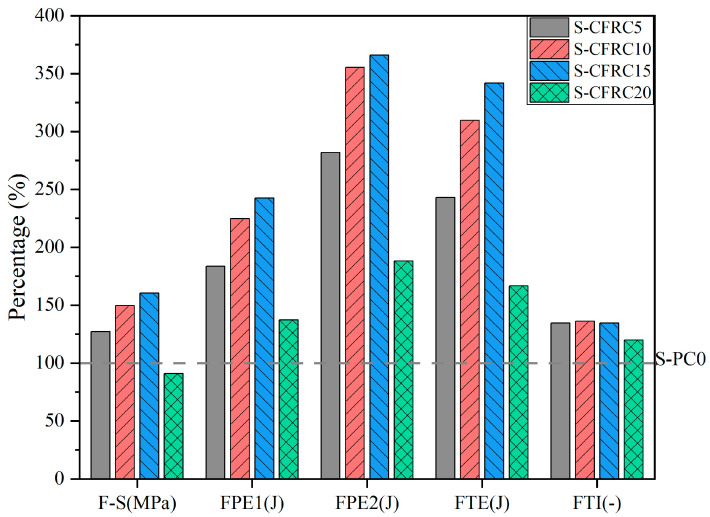
Effect of matrix modification on CFRC flexural properties [103].

Recent research has shown that sisal fibers added to a cement matrix can increase the flexural strength of hardened concrete by up to 58% [142]. The incorporation of steel fibers, palm fibers and blended synthetic fibers into a concrete matrix has been demonstrated to significantly enhance the flexural properties of concrete [143]. When 0.25% palm fiber and 1.75% steel fiber were incorporated, the flexural properties of concrete were improved by 42.5% compared to the concrete without fiber [143]. It has been shown that the incorporation of wood fibers enhances flexural strength significantly [144]. However, it is important to note that the reason for enhancement may be the low strength of the matrix [144]. Similar to compressive strength, common measures to improve the flexural properties of PFRCCs also include matrix modification and fiber modification. As shown in Figure 14, compared with untreated jute fibers, alkali modification of jute fibers followed by incorporation into the matrix resulted in a significant improvement in the flexural properties of JFRC [27]. As illustrated in Figure 15, substituting silica fume for cement led to improvements in flexural strength (F-S), pre-crack energy absorption (FPE1), post-crack energy absorption (FPE2), total energy absorption (FTE) and the flexural toughness index (FTI) [103].

According to the study, the model of the flexural strength of CFRC is shown in Equations (7) and (8) [134].

(7) $$f_{fs14}=4.25+0.089X_{1}+1.61X_{3}+1.99X_{2}-1.1X_{3}^{2}-1.26X_{2}^{2}-0.866X_{2}X_{3}$$

(8) $$f_{fs28}=1.88+0.098X_{1}+1.64X_{3}+2.22X_{2}-1.16X_{3}^{2}-1.37X_{2}^{2}-0.966X_{2}X_{3}$$

where *f_fs_*_14_ and *f_fs_*_28_ are the flexural strength at 14 days and 28 days and *X*_1_, *X*_2_ and *X*_3_ are jute, coconut fiber and quarry dust, respectively.

The tensile stress produced by bending moments is converted to sheering stress at the interface between the PF and matrix when the PFRCC is subjected to bending loads. This shear stress is counteracted by the mechanisms of adsorption and friction present at the contact surface [145]. In addition to impacting the component interface, this contact force also exerts its influence within the cement matrix at a location distant from the interface [145]. Consequently, an annular region encircling the fibers is established through the combined influence of both the PF and the matrix [146]. This is similar to the principle of reinforced concrete. The plant fiber in the composite material is like the rib of a steel bar used to disperse tensile force, thereby increasing the bearing capacity of the section.

### 4.3. Tensile Behavior of PFRCCs

**Figure 16:**
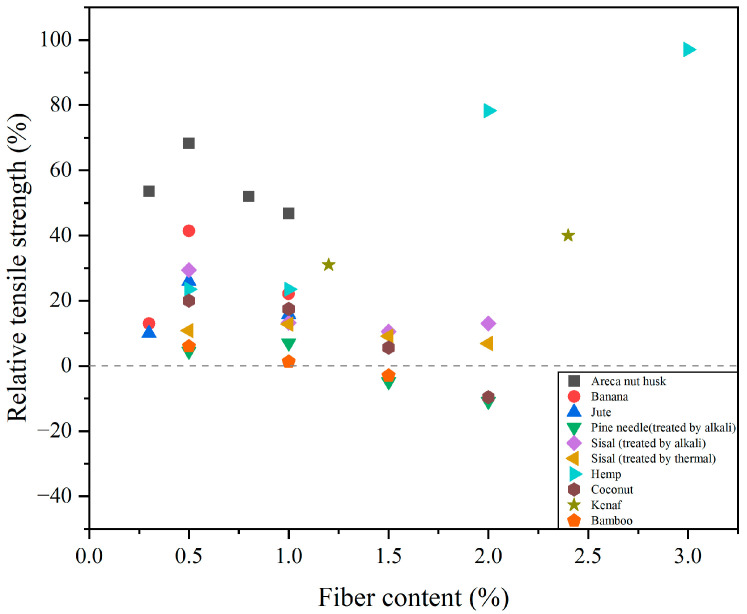
Impact of PF on relative tensile strength of PFRCCs.

In general, CCs have low tensile strength, and it is crucial to determine when cracks appear. The load of CC cracking affects the appearance and development of cracks in other parts [147]. Studies have shown that adding PF to CCs can reduce crack width, prevent crack development and indirectly increase tensile strength [148]. The primary factors influencing the tensile strength of PFRCCs are the elastic modulus and the tensile strength of the PF used. As shown in Figure 16, different researchers have studied the tensile properties of PFRCCs to which different PFs have been added [28,42,64,106,138,141,149,150]. According to Laborel-Préneron et al. [38], the addition of 1.5% coir fiber can significantly improve the splitting tensile strength of PFRCCs. In another study, the researchers observed that incorporating 0.2% coconut fibers resulted in a 22.9% increase in splitting tensile strength compared to plain concrete [123]. The surface of coconut fiber is rough, which improves the interface adhesion between the fiber and the cement matrix. Studies by other authors on banana fibers, jute fibers and masson pine fibers have also shown that the incorporation of appropriate PFs has a positive effect on the splitting tensile strength of concrete [42,138,149].

Sultana et al. established a model of the relationship between jute fiber length, fiber volume, water–cement ratio and tensile strength based on the response surface method. As indicated in Equation (9) [133], fiber content emerges as the primary determinant influencing tensile strength [133]. Mathematical models for predicting the tensile strength of jute and bamboo fiber-reinforced silica fume concrete, also based on the response surface method, are shown in Equations (10) and (11) [134]. The relationship between the tensile strength and compressive strength of PFRCCs is not a simple linear correlation. Compared with the compressive strength, the incorporation of plant fiber results in a greater increase in tensile strength. The relationships between banana fiber volume (*V_f_*) and compressive strength (CS) and splitting tensile strength (STS) are shown in Equations (12) and (13) [138]. Wang et al. obtained the relationship between STS, age of maintenance (t) and corn fiber content (ω) using Design-Expert software, as shown in Equation (14) [34]. Based on the equation, it is obvious that the STS of corn fiber-reinforced CCs exhibits a negative correlation with curing age.

(9) $$\begin{array}{l} f_{t}=2.57+0.035L-0.203V-0.01W/C-0.014L^{2}+0.242V^{2}\\ +0.032W/C^{2}+0.434LV-0.163LW/C+0.154VW/C \end{array}$$

where *f_t_* is the tensile strength of JFRC.

(10) $$\begin{array}{l} f_{sts14}=2.280+0.0246X_{1}-0.0983X_{3}+0.040X_{2}-0.09X_{3}^{2}\\ -0.155X_{2}^{2}-0.041X_{2}X_{3} \end{array}$$

(11) $$\begin{array}{l} f_{sts28}=2.65+0.0235X_{1}-0.093X_{3}+0.091X_{2}-0.102X_{3}^{2}\\ -0.218X_{2}^{2}-0.072X_{2}*X_{3} \end{array}$$

where *f_sts_*_14_ and *f_sts_*_28_ are the splitting tensile strength of concrete at 14 days and 28 days, respectively, and *X*_1_*, X*_2_ and *X*_3_ are jute, coconut fiber and quarry dust, respectively.

(12) $$STS=(-0.352*V_{f}^{2}+0.462*V_{f}+0.408)\sqrt{CS}(For-BSFRC-28days)$$

(13) $$FTS=(-0.339*V_{f}^{2}+0.282*V_{f}+0.83)\sqrt{CS}(For.BSFRC-28days)$$

(14) $$\begin{array}{l} STS=1.17+0.13t-0.49\omega -0.2\omega t-0.33t^{2}+0.02\omega^{2}\\ +0.24t^{2}\omega +0.11\omega^{2}t+0.3t^{3}+0.07\omega^{3}(R^{2}=0.9715) \end{array}$$

However, it should be noted that current studies show that there is a threshold of PF incorporation in CCs [19,148]. According to these studies, the main reasons for the reduction in the tensile strength of composites caused by excessive fiber incorporation are as follows: (1) The addition of fiber reduces the porosity of the concrete matrix, which has a negative effect on the static tensile strength of the PFRCC. (2) An excessive amount of fiber can significantly reduce the fluidity of the mixture, which in turn causes an uneven distribution of PF within the matrix. This phenomenon ultimately contributes to a decrease in the tensile strength of the PFRCC.

### 4.4. Elasticity Moduli of PFRCCs

**Figure 17:**
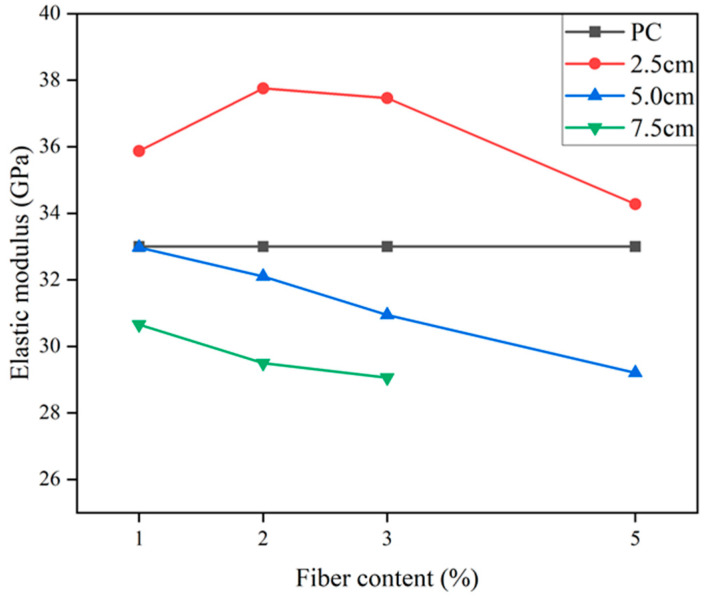
Effects of length and content of coconut fibers on the elastic modulus of CFRC.

According to a large number of studies, different physical friction levels and lengths of PFs have different effects on Young’s modulus values [123]. Some researchers have studied the effect of coconut fiber on elastic modulus. The results showed that the elastic modulus of corn fiber-reinforced concrete (CFRC) increased by 15% compared with plain concrete [39]. In fact, different fiber dosages and fiber lengths will have different effects on the modulus of elasticity of a PFRCC, as shown in the following Figure 17 [39]. The test results show that 25 mm coir fiber causes the greatest enhancement in the modulus of elasticity of a CFRC, while 50 mm and 75 mm coir fibers negatively affect the modulus of elasticity of the CFRC regardless of the dosing level. Also, the study developed an equation for estimating the static modulus of elasticity, as shown in Equation (15) [39].

(15) $$E_{static}=X_{s}+Y_{s}c+Z_{s}c^{2}$$

where *c* is the fiber content parameter of the values and *X_S_*, *Y_S_* and *Z_S_* are constants corresponding to coconut fiber length.

Other scholars have investigated the impact of hybrid fiber (steel fibers and palm fibers) on the elastic modulus of high-flowing concrete [143]. The research indicated that incorporating palm fiber in conjunction with steel fiber enhances the elastic modulus of high-flowing concrete. The observed increase in the modulus of elasticity can be primarily attributed to the high stiffness of steel fiber [143]. However, the researchers found that the static moduli of CCs were reduced when the fiber volume fraction was more than 1%. This may be because the elastic modulus of a CC is greatly affected by the volume fraction of fiber. Some researchers have studied the effect of industrial hemp fibers on the elastic modulus of concrete [129]. The results showed that the elastic modulus was improved with the addition of hemp fiber [129]. The elasticity modulus can be used to determine the dimensions of non-reinforced structures. The results further proved that adding industrial hemp fiber can enhance the ductility of concrete [129]. All the above studies have proven that the appropriate amount of fiber is beneficial to the elastic modulus.

### 4.5. Fracture Behavior of PFRCCs

Because of the high tensile strength and small diameter of PFs, the interface bonding between PFs and CCs is strong [151]. Certain PFs exhibit a fine size and possess a substantial specific surface area, which leads to increased chemical bonding within the interfacial transition zone (ITZ) [152]. Concurrently, the surface roughness of the PF contributes to an effective combination with the CC. However, the distribution of PFs in CCs is random. During the stirring process, some PFs may not disperse evenly but join together in clumps or bundles [153]. The uneven distribution of PFs causes some fibers to fail to prevent the spread of cracks [67].

**Figure 18:**
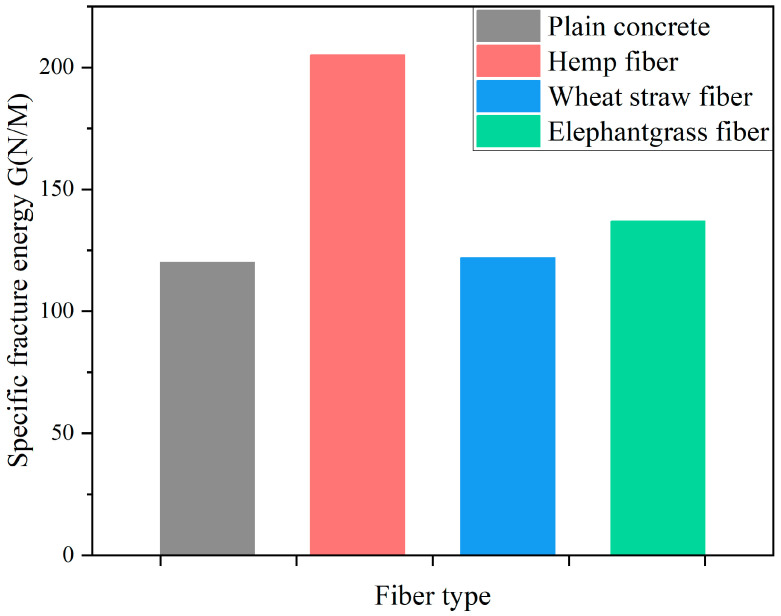
Effects of different PFs on fracture energy [153].

**Figure 19:**
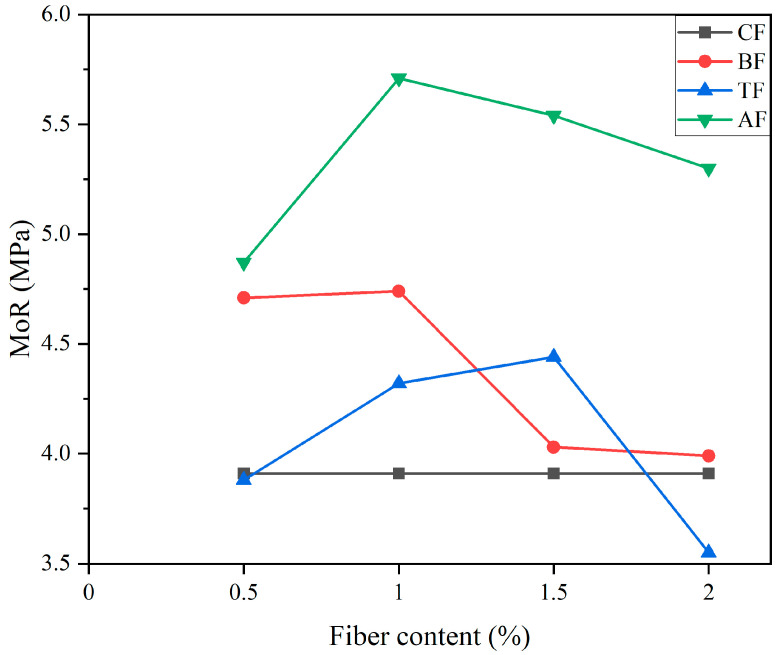
Effects of pretreatment methods and contents of pine needle fiber on MoR [42].

Merta and Tschegg [153] conducted an investigation into the fracture performance of concrete that had been reinforced with hemp fiber, elephant grass fiber and wheat straw fiber. The methods of uniaxial testing and wedge splitting were adopted. As shown in Figure 18, the findings indicated that the incorporation of PF enhanced the specific fracture energy of the concrete [153]. Hemp fiber exerted a substantial influence on fracture performance, resulting in a 70% enhancement in fracture energy. This phenomenon can be primarily attributed to the fine structure of hemp fibers, which have a diameter ranging from 16 to 50 μm, along with their considerable specific surface area. Consequently, hemp fibers and cement matrices exhibit strong bonding strength [153]. In the research, the hemp fibers were observed not to fracture, but to pull out along the fracture surface. It has been shown that pretreated PF can enhance the modulus of rupture (MoR) of PFRCCs [42]. As shown in Figure 19, the MoR results of the experimental samples treated with alkali (AF) and boiling water (BF) were greater than those of the control group (CF), except for the 0.5% and 2% contents of pine needle fibers after a soaking treatment (TF) [42].

### 4.6. Dynamic Mechanical Properties of PFRCCs

Some scholars have paid attention to the tensile, compressive and bending properties of PFRCCs under dynamic loads. Ahmed and Ali [154] studied the effect of jute fiber on the impact properties of concrete. The specific goal of the researchers was to improve the impact resistance of concrete walls by adding jute fibers [47]. An improved pendulum impact device was used for the test [155]. The findings demonstrated that jute fiber added to concrete performs exceptionally well in terms of impact resistance. From the research conducted in recent years, flax fiber is widely used to enhance the impact resistance of concrete [155,156]. The impact properties of flax fiber-laminated sheet-reinforced beams were investigated, and the results showed that the impact resistance of flax fiber laminates increased with their thickness, but the bonding properties between flax fiber laminates and concrete need attention [156]. Wang et al. investigated the dynamic compression characteristics of flax fiber-reinforced concrete under impact loading and derived the relationship between the strain rate and the dynamic influence factor (DIF) of compressive strength, as shown in Equations (16) and (17) [157].

(16) $$DIF=0.731\mathrm{lg}\overset{•}{\varepsilon }-0.21,50\leq \overset{•}{\varepsilon }\leq 200S^{-1}$$

(17) $$DIF=1.1043e^{0.0513\overset{•}{\varepsilon }},0.2\leq \overset{•}{\varepsilon }\leq 30S^{-1}$$

where $\overset{•}{\varepsilon }$ is the strain rate.

Zhang et al. [148] proposed an advanced predictive model for the dynamic increase factor of tensile strength in PFRCCs. Their study investigated high-strength concrete reinforced with sisal fibers of different lengths. The dynamic tensile strength of specimens was evaluated using the split Hopkinson pressure bar test [71]. To quantitatively assess the strain rate sensitivity of ultra-high-performance concrete (UHPC), linear fitting was applied to the dynamic tensile performance data for six varieties of UHPC specimens [148]. The findings indicated that the length of sisal fibers significantly influenced the strain rate response of the UHPC specimens [148]. A critical factor contributing to the improved dynamic tensile strength in fiber-reinforced concrete is the ability of these fibers to mitigate microcrack formation during the initial stages of damage [158]. As stress levels increased, the microcracks developed into larger fractures. During this process, the short fibers nearby were gradually extracted, while the long fibers predominantly handled the load. These factors contribute to the higher energy demands for the failure of UHPC reinforced with sisal fibers [158].

In addition to the above impact tests, other scholars carried out dynamic properties tests on the component levels of PFRCCs. Both the damping ratios and fundamental frequencies of plant fiber-reinforced CCs were studied in recent research [39,154]. In the research of Ali et al. [39], as expected, the effect of plant fiber on dynamic properties before injury was not obvious compared with that during injury. After cracking, the damping ratio increases and the fundamental frequency decreases. Another study on PFRCC walls supports this argument [154]. In general, the incorporation of PF into a cement matrix enhances the impact strength and dynamic tensile modulus and increases damping ratios. Therefore, it can be concluded that PFs have the potential to be applied in reinforced concrete and that such reinforcement can continue to be practiced in construction projects.

### 4.7. Impact Factors of Plant Fiber in PFRCCs

The influence of fiber type, fiber length and fiber content on the mechanical properties of PFRCCs can be described in the following manner. Based on the fiber spacing theory, the average distance between fibers regulates their reinforcing influence on CCs. A reduced fiber spacing enhances the reinforcing capability of fibers within a matrix [159]. This enhancement is associated with several factors, including the surface roughness of the fibers, the adhesion between the fibers and the matrix, the content of fibers, the length of the fibers, and their distribution throughout the matrix.

Research has already revealed that there is a strong relationship between rheological properties and mechanical properties [160,161]. This relationship also exists in PFRCCs. Sawsen et al. [162] researched the rheological and mechanical properties of flax fiber-reinforced CCs. The results illustrated that the incorporation of pretreated flax fiber exhibited a positive effect on the rheological and mechanical properties [162]. These results are supported by Gwon and Shin, who argued that the rheological properties and mechanical properties exhibited the same trend with the incorporation of cellulose microfibers [163]. The characteristics of PF are influenced by its source, extraction technique and processing, which in turn impact the performance of PFRCCs. The formation of PF by extrusion, injection molding or compression molding can effectively reduce the influence of moisture on it [164]. Fabrics composed of PF are widely employed in cement-based gel composites. One of the most effective techniques for obtaining high-performance cement-based gel composites is reinforcement with fabrics (textiles). Such systems have a superior fiber–matrix bonding, which improves mechanical properties more than continuous or staple fibers [165]. According to the research of Peled and Bentur [165], on the one hand, the geometry of a given plant fiber fabric can enhance the adhesion of the PF to the substrate; on the other hand, the geometry of the fabric can significantly reduce the possibility of uneven distribution of the fibers in the substrate [166]. Another common PF form in concrete is paper pulp, the application of which promotes uniform distribution [32]. Experimental results showed that cellulose pulp is a good micro-reinforcer for cement-based gel composites at the early stage [32]. Compared to the use of monofilament fibers, pulp is stable in multiple directions and therefore can enhance properties in multiple directions. However, it should be noted that, in practice, pulp often has insufficient embedding length, which affects the bridging effect of the fibers.

The characteristics of PFs greatly influence the mechanical properties of PFRCCs, including the water content of the fibers, their length-to-diameter ratio and their orientation. When water is absorbed, it disrupts the hydrogen bonds either between the fibers or between the fibers and the matrix, resulting in a notably weak bond between PFs that have high water absorption and the matrix [167]. The mechanical characteristics of PFRCCs are also influenced by their aspect ratios. Experiments have shown that a low length–diameter ratio is better for fiber pull-out and that a high length–diameter ratio helps to enhance performance in multiple directions [167]. Additionally, the alignment of the fibers influences the mechanical characteristics. Research indicates that the bending and tensile strength in alignment with the direction of the plant fibers is greater than that observed in the lateral orientation [168].

## 5. Interfacial Bonding Properties of PFRCCs

**Figure 20:**
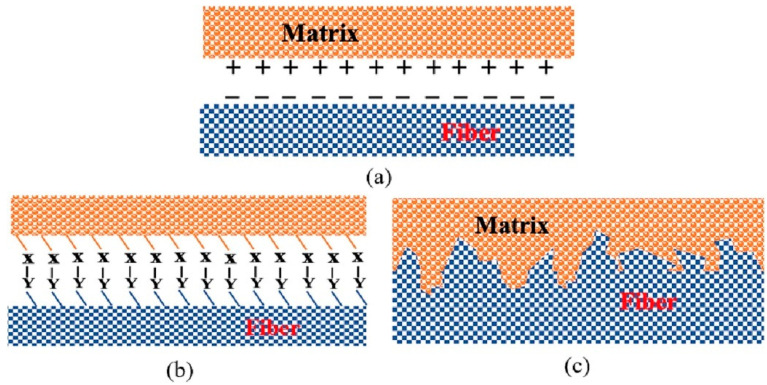
Methods of bonding between matrix and fiber: (**a**) electrostatic attraction; (**b**) chemical bonding; (**c**) mechanical interlocking [169].

Over the years, the concept of an ITZ or “transition halo” around fine and coarse aggregates in concrete has become an accepted principle in concrete technology [152]. In FRCCs, the properties of the ITZ affect the stress transfer between the fiber and the cement matrix, which is the key parameter to determine the properties of an FRCC [169]. As shown in Figure 20, the bonding mechanism between PF and a CC mainly includes electrostatic attraction, chemical bonding and mechanical interlocking [170]. In general, due to the different adhesion of the fiber–matrix interface, several adhesion mechanisms can function simultaneously.

### 5.1. Measurement of Interface Bonding Parameters

**Figure 21:**
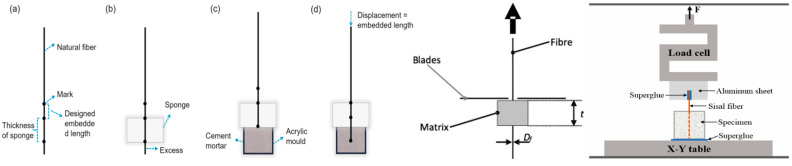
Commonly used single-fiber pull-out test: (**a**) Stage 1; (**b**) Stage 2; (**c**) Stage 3; (**d**) Stage 4 [26,67,111,151,171].

Micromechanical testing is used to evaluate the characteristics of bonding properties between PF and a CC. These measurements enable a clear understanding of load distribution and failure mechanisms occurring at the ITZ. The micromechanical method commonly used to test the bonding properties of PFRCCs is the pull-out test, as shown in Figure 21 [26,67,111,151,171].

The bonding strength between PF and a CC can be determined using the single-fiber pull-out test, which has seen much development and improvement in recent years. Ren et al. [67] investigated the bonding properties of sisal fibers with UHPC by the pull-out test. In the experiment, fibers were first passed through the center of the side of the mold using a needle and tweezers, and then the mixture was poured into a cubic silicone mold. Zhao et al. [26] straightened and marked the selected fibers using hanging clips, then used a needle to pass the fibers vertically through the sponge to ensure that the fibers were embedded vertically in the matrix. Lecompte et al. [151] uses two blades to block the matrix, thereby shearing the interfacial region and applying tension to the fibers. In contrast to the single-fiber pull-out test described above, the multifiber pull-out test will not exclude fibers that have failed to stretch, which makes the results of the multifiber pull-out experiment closer to the true pull-out behavior of fibers [30].

**Figure 22:**
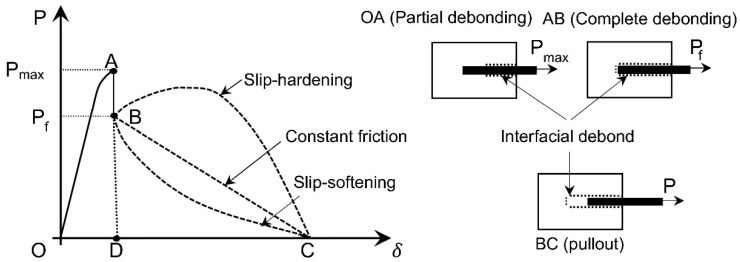
Schematic diagram of typical pull-out curves and related pull-out mechanisms [171].

The standard pull-out curves of PF from a cement matrix are illustrated in Figure 22 [171]. In this curve, the OA segment is the initial phase; the tensile stress is transferred from the plant fiber to the gelling matrix through a combination of chemical and mechanical interactions between the fiber and the matrix. The load at point A is called the critical pull-out load. Section AB is the complete stripping stage, which indicates the transition from chemically and physically controlled debonding to physically controlled pull-out. Segment BC is the pull-out stage, where any of the following three mechanisms can occur: (a) constant slip, (b) slip hardening and (c) slip softening (as shown Figure 22), which depends mainly on the nature of the friction interface [151]. The researchers obtained bonding strength (τ), pull-out energy ($G_{w}$), interfacial sliding shear strength ($\tau_{f}$), apparent interfacial shear strength (IFSS) and pull-out strength ($f_{p}$) values according to the peak pull-out stress and the bond area between the PF and the cement matrix, as shown in Table 6 [30,67,151,171].

### 5.2. Interface Microstructure Test

To analyze the chemical composition distribution and roughness of the interface between plant fibers and a cement-based gel matrix, researchers commonly employ a variety of physical and chemical observation techniques. Commonly used methods include SEM, combined energy dispersive spectroscopy (EDS) and acoustic emission (AE) [26,30,34,67,111,171,172,173]. The following Table 7 summarizes the characterizations of ITZ by different researchers.

**Figure 23:**
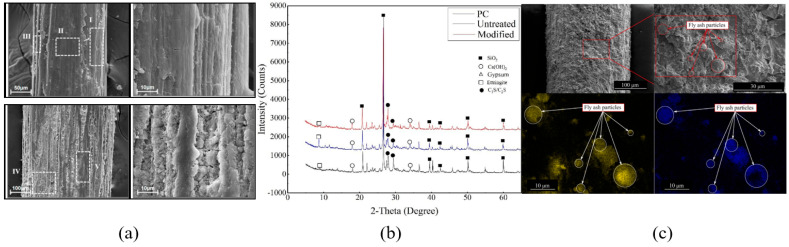
(**a**) SEM images of fiber surfaces after being pulled out from cement matrix; (**b**) the XRD patterns of RSFRC; (**c**) pulled-out coir fibers under SEM and EDX [26,30,111].

Different components in composites at the microscale can be observed by SEM. SEM is extensively employed to observe the ITZs of PFRCCs. Additionally, to determine the distribution of each element in an SEM image, EDS is typically employed in conjunction with SEM. Researchers used SEM and EDS techniques to analyze the surface appearance of sisal fibers coated with SCA and NS when dispersed in a matrix [26]. Researchers chose to perform EDS within the white-marked area in Figure 23a to obtain the chemical composition of each element on the plant fiber surface [26]. The findings demonstrated that SCA and NS encapsulation increased the amounts of Si and Ca on the sisal fiber surface, indicating a strong interaction between the matrix and the PF [26]. Some researchers performed X-ray diffraction analysis of nano-SiO2-modified straw fiber-reinforced concrete (RSFRC), as shown in Figure 23b [111]. The results indicated that the incorporation of nano-SiO2 increased the yield of silica–calcium hydroxide and decreased the yield of ettringite, which enhanced the structural density of the ITZ [111]. Wang et al. [30] used SEM and EDS techniques to analyze the pull-out of coconut fibers in mortar, as shown in Figure 23c. The findings demonstrate that the fiber–matrix interface bonding strength cannot be strengthened by the fly ash pozzolanic process. This is because, during the pull-out operation, the unreacted spherical fly ash particles on the fiber surface act as lubricants [30].

### 5.3. Theoretical Model of Pull-Out Behavior

The ITZ is crucial to the mechanical performance of PFRCCs, since it is the area where forces are passed from the matrix to the fibers [86]. A number of models for the bonding behavior at the fiber–matrix interface have been studied by researchers over the past few decades. The earliest model of pull-out behavior was proposed by Cox [175]. The model ignores the shear stresses in the fibers and models all mechanics as elastic. The positive stresses in the fibers ($\sigma_{f}$) and the shear stresses at the interface ($\tau_{i}$) obtained from Cox’s model are shown in the following Equations (18) and (19) [175].

(18) $$\sigma_{f}=E_{f}\varepsilon_{f}\left[1-\cosh(nx/r)/\cosh(ns)\right]$$

(19) $$\tau_{i}=nE_{f}\varepsilon_{l}\sinh(nx/r)/2\cosh$$

where *E_f_* is the fibers’ Young’s modulus, *ε_l_* and *ε_f_* are the strain of PF, *s* is the fibers’ aspect ratio, *n* is a constant, *r* is the variation rate of the matrix and *x* is the length variable of the fibers (0 to l).

In later research work, Ferreira et al. [173] formulated a model for simulating the interaction of PF and a matrix during processes. They assumed that the fibers were linearly elastic and perfectly rigid and that the interaction between the matrix and the fibers was based on the bond-slip law [173]. The model assumes a bilinear bond-slip law, as shown in the following Equation (20) [173].

(20) $$\tau (s)=\left\{\begin{array}{l} k_{el}*s\\ \tau_{r}-k_{in}*(s-s_{el})\\ 0 \end{array}\right.\begin{array}{c} if\\ if\\ if \end{array}\begin{array}{c} \left|s\right|\\ s_{el}\\ \left|s\right| \end{array}\begin{array}{c} \leq \\ <\\ > \end{array}\begin{array}{c} s_{el}\\ \left|s\right|\\ s_{u} \end{array}\begin{array}{cc} \leq & s_{u} \end{array}$$

where $k_{el}=\tau_{\max}/s_{el}$ is the elastic branch of the sliding modulus, *τ_r_* is the residual bond stress, *k_in_* is the post-peak sliding modulus (strictly positive) and *s_u_* is the slip distance.

## 6. Conclusions

This review synthesizes advancements in PFRCCs, identifying critical knowledge gaps and proposing transformative pathways for future research. The following scientific insights emerge as pivotal to advancing the field:

(1) The four chemical constituents of PFs are cellulose, hemicellulose, lignin and pectin. While the hierarchical structure of PFs (cellulose, hemicellulose, lignin and pectin) provides intrinsic mechanical advantages, their hygroscopicity and alkaline degradation remain unresolved contradictions in cement matrices.
(2) Modification methods include the modification of matrices and PFs. The alkalinity of a matrix can be effectively decreased by substituting different cement-based gel materials for cement, either whole or partially. However, the review uncovered a critical oversight: current studies predominantly focus on short-term mechanical gains while neglecting carbonation-accelerated fiber mineralization.
(3) The appropriate amount of PF encourages hydration by removing water from the cavities and enhances mechanical properties and fracture behavior through bridging effects. It is worth noting that the compressive strength of CFRCCs is decreased by most kinds of plant fiber. It is essential to show how the ITZ affects the mechanical properties of PFRCCs.
(4) Life cycle analyses indicate that current PFRCC formulations achieve only 12–18% embodied carbon reduction compared to steel fiber-reinforced cementitious composites, underscoring the urgency of developing low-pH cement-based gel composites compatible with plant fibers.

In general, a critical knowledge gap persists in elucidating the degradation mechanisms of PF within PFRCCs. Concurrently, advanced surface modification strategies must be engineered for both PF and CCs to improve the mechanical properties and durability of PFRCCs.

## Figures and Tables

**Table 1:** Chemical compositions of different PFs.

Plant fiber	Cellulose (%)	Hemicellulose (%)	Pectin (%)	Lignin (%)	Ref.
Abaca	62.5	21	0.8	12	[57]
Alfa	45.4	38.5	-	14.9	[58]
Bagasse	37	21	10	22	[57]
Banana	62.5	12.5	4	7.5	[59]
Bamboo	34.5	20.5	-	26	[60]
Coir	46	0.3	4	45	[61]
Cotton	89	4	6	0.75	[62]
Flax	72.5	14.5	0.9	2.5	[57]
Hemp	74.4	17.9	3.7	1.7	[61]
Jute	67	16	0.2	9	[63]
Kenaf	53.5	21	2	17	[64]
Pineapple	80.5	17.5	4	8.3	[62]
Ramie	72	14	2.0	0.8	[62]
Sisal	60	11.5	1.2	8	[65]

**Table 2:** Mechanical properties of plant fibers [52].

Fiber Type	Relative Density (g/cm^3^)	Tensile Strength (MPa)	Elastic Modulus (GPa)	Specific Modulus (GPa·cm^3^/g)	Elongation at Failure (%)
Abaca	1.5	400–980	6.2–20	9	1.0–10
Bamboo	0.6–1.1	140–800	11–32	25	2.5–3.7
Banana	1.35	500	12	9	1.5–9
Coir	1.15–1.46	95–230	2.8–6	4	15–51.4
Cotton	1.5–1.6	287–800	5.5–12.6	6	3–10
Flax	1.4–1.5	343–2000	27.6–103	45	1.2–3.3
Hemp	1.4–1.5	270–900	23.5–90	40	1–3.5
Jute	1.3–1.49	320–800	30	30	1–1.8
Ramie	1.0–1.55	400–1000	24.5–128	60	1.2–4.0
Sisal	1.33–1.5	363–700	9.0–38	17	2.0–7.0

**Table 4:** Pros and cons of different plant fiber modification methods.

Modification Methods	Cons	Pros	Refs.
Alkali treatment	Simple to operate	Has the risk of damaging the strength of PF	[113,114]
Water retting	Benefit for the interface between PF and cement matrix	Consumes water and contributes to water eutrophication	[115]
Plasma treatment	Modifies surface of PF without affecting the bulk properties		[116]
Thermal treatment	Simple to operate	Has the risk of leading to thermal degradation of PF	[117]
Acetylation treatment	Exhibit excellent capacity to remove the defects on the surface of PF		[114]

**Table 5:** Recommended dosages of plant fibers for improving compressive strength.

Fiber Type	Recommended Dosage Range (%)	Matrix	Ref.
Sisal	1–3	Concrete	[65]
Flax	0.1–0.2	Concrete	[29]
Coconut	1–2	Concrete	[39]
Hemp	0.5	Concrete	[129]
Jute	0.2–0.4	Mortar	[27]
Banana	0.1–0.25	Concrete	[71]
Date palm	0.5	Mortar	[33]

**Table 6:** Main parameters of pull-out performance.

Mechanical Parameter	Formula
Bonding strength (τ)	$\tau =\frac{P_{\max}}{\pi Cl}$
Pull-out energy ($G_{w}$)	$G_{w}=\frac{\int_{0}^{l}p(s)ds}{\pi Cl}$
Interfacial sliding shear strength ($\tau_{f}$)	$\tau_{f}=\frac{P_{\max}}{\pi d_{e}l}$
Apparent interfacial shear strength (IFSS)	$IFSS=\frac{F_{bond}}{\pi tD_{f}}$
Pull-out strength ($f_{p}$)	$f_{p}=\frac{P_{\max}}{\sum_{i=1}^{30}(\pi \bar{d_{i}}l)}$

*P_max_* means the maximum pull-out load; *C* means the section girth of plant fiber; *l* means the initial embedded length; *s* is the slip of plant fiber; *τ_f_* means interfacial sliding shear strength; *d_e_* is the equivalent fiber diameter; *F_bond_* is the first maximum force; *t* and *D_f_* are, respectively, the matrix thickness and fiber diameter; *f_p_* is the pull-out strength; $\bar{d_{i}}$ is the average diameter of fiber.

**Table 7:** Characterization of PFRCC interfacial transition zone properties.

Property	Method	Feature	Applications in Interface Analysis	Ref.
Micromechanical performance	Acoustic emission	Strength of the interface zone	The intensity of the acoustic emision signal represents the difficulty of disrupting the ITZ	[34]
Observation of interphase	SEM	Microstructural morphology	Investigation of the interfacial characteristics between sisal fibers and the cementitious matrix	[67]
Observation of interphase	SEM	Microstructural morphology	Investigation of the effect of surface-coated silane coupling agent (SCA) and nano-SiO_2_ (NS) on the interfacial bonding ability of sisal fibers in cementitious composites	[26]
Micromechanical properties	Nanoindentation	Hardness and indentation modulus of the ITZ	It was examined how internal curing affected the fiber–matrix inter-face region’s microstructure	[174]
Interphase composites	EDS	Determination of the material’s composition on the fiber surface	Identifying fiber pull-out patterns by examining whether mortar particles are present on the fiber surface	[171]
Observation of interphase	SEM	Microstructural morphology	Investigating the effect of different pH substrates on plant fiber degradation as well as ITZ	[30]

## Data Availability

Data sharing is not applicable to this article as no new data were created or analyzed in this study.
